# Sámi reindeer herders’ perspective on herbivory of subarctic mountain birch forests by geometrid moths and reindeer: a case study from northernmost Finland

**DOI:** 10.1186/s40064-015-0921-y

**Published:** 2015-03-20

**Authors:** Terhi Vuojala-Magga, Minna T Turunen

**Affiliations:** Arctic Centre, University of Lapland, POB 122, FI-96101 Rovaniemi, Finland

**Keywords:** Reindeer herding, *Betula pubescens* ssp. czerepanovii, Mountain birch destruction, Autumnal moth, Winter moth, Climate change, Global warming, Indigenous knowledge (IK), Professional knowledge, Niche construction theory (NCT)

## Abstract

**Introduction:**

Geometrid moths and semi-domesticated reindeer are both herbivores which feed on birch leaves in the subarctic mountain birch forests in northern Fennoscandia. The caterpillars of autumnal and winter moths have episodic outbreaks, which can occasionally lead to extensive defoliation of birch forests. Earlier studies have shown that reindeer have a negative effect on the regeneration of defoliated birches by grazing and browsing their seedlings and sprouts.

**Case description:**

We interviewed 15 reindeer herders in the Kaldoaivi and Paistunturi herding co-operative in northernmost Finland in order to analyse their past, present and future views on the behaviour of moths and the growth of mountain birches. We investigate the behaviour of the two herbivores by combining the indigenous knowledge (IK) of Sámi herders with the results of relevant studies in biology and anthropology, applying niche construction theory (NCT) in doing so.

**Discussion and evaluation:**

In the first stage, the niche constructors (moths, reindeer, herders, mountain birch and other organisms) are looked upon as “equal constructors” of a shared niche. As changes unfold in their niche, their role changes from that of constructor to key constructor. The role and importance of niche constructors were different when nomadic pasture rotation was used than they are today under the herding co-operative system. Niche construction faced its most radical and permanent negative changes during the border closures that took place over the latter half of the 19^th^ century. The large-scale nomadic life among the Sámi herders, who migrated between Finland and Norway, came to an end. This phase was followed by stationary herding, which diminished the possibilities of reindeer to look for various environmental affordances. Difficult snow conditions or birch defoliation caused by moth outbreaks made the situation worse than before. Eventually reindeer became key constructors, together with moth larvae, leading to negative ecological inheritance that forced herders to use new, adaptive herding practices.

**Conclusions:**

Both the scientific data and the IK of herders highlight the roles of reindeer and herders as continuous key constructors of the focal niche, one which stands to be modified in more heterogenic ways than earlier due to global warming and hence will result in new ecological inheritance.

## Introduction

Reindeer herding is one of the oldest livelihoods in northern Fennoscandia (Kortesalmi 2007:22–23). Starting in the 1600s, Northern, or Fell, Sámi families roamed hundreds of kilometres with their herds from summer pastures on the coast to winter pastures inland and back again. In Finland, reindeer herding has experienced a number of changes, including the adoption of an organized system of fenced co-operatives, a shift from a subsistence to a financial economy, a transition to permanent housing, modernization in technology and changing herding systems (Kortesalmi 2007; Helle and Jaakkola 2008; Vuojala-Magga et al. 2011; Turunen and Vuojala-Magga 2014). The interplay between ecological, historical, economic and political factors affecting reindeer herding in the northernmost part of Fennoscandia has been discussed in the literature (Lehtola 2004, 2012; Lempa et al. 2005; Heikkinen 2006; Caballero et al. 2007; Näkkäläjärvi 2007, 2013; Tyler et al. 2007; Helander-Renvall 2008, 2014; Riseth and Vatn 2009). Mountain birch (*Betula pubescens* ssp. czerepanovii (Orlova) Hämet-Ahti), a hybrid of dwarf (*B. nana*) and downy birch (*B. pubescens*), is an important plant for the reindeer herding communities in northern Fennoscandia (Wielgolaski 2005; Holtmeier 2007). In early summer, when female reindeer are lactating, young birch leaves serve as a principal source of nutrition for reindeer (Warenberg et al. 1997). In mountain birch forests, practically all large-sized mushrooms are mycorrhizal fungi of birch (Ruotsalainen et al. 2009). Mushrooms are the animals’ preferred forage in autumn. Mountain birch forests are also important in winter as they support epiphytic lichens, which offer forage, particularly when digging conditions for ground lichens are difficult. Birches also provide shelter for people: logs and branches are used for building *laavus* (lean-tos) and the wood is used for heating and cooking. In winter birch forests also serve as avalanche barriers.

The value of Sámi reindeer herders’ professional knowledge and/or indigenous knowledge (IK) has been acknowledged particularly when herding communities have been subjected to uncertain conditions (Tyler et al. 2007; Saijets and Helander-Renvall 2009; Riseth et al. 2010; Vuojala-Magga et al. 2011; Vuojala-Magga 2012; Syrjämäki and Mustonen 2013; Helander-Renvall 2014). Although reindeer herding has been one of the key activities in the mountain birch forests, there are only few studies in which Sámi herders’ IK has been integrated with scientific studies in biology, and particularly few in which the interaction between geometrid moths, mountain birches and reindeer grazing has been examined (Rybråten and Hovelsrud 2010). The caterpillars of autumnal and winter moths have episodic outbreaks which can occasionally lead to extensive defoliation of birch forests in Northern Fennoscandia (Tenow 1972; Kallio and Lehtonen 1975; Lehtonen and Heikkinen 1995; Holtmeier et al. 2003). Regeneration of these forests greatly depends on the environmental conditions, including the extent of reindeer grazing (Lehtonen and Heikkinen 1995; Helle 2001, den Herder and Niemelä 2003; Cairns and Moen 2004). We apply niche construction theory (NCT) to combine indigenous knowledge (IK) of Sámi herders from two herding co-operatives in northernmost Finland - Kaldoaivi and Paistunturi – with the results of scientific studies in biology. This approach enables us to analyse the changes in the niche from the perspective of herders. The key questions are: What is the importance, as viewed through NCT, for the reindeer herding environment of birch defoliation caused by moth outbreaks? How have the niche and its tempo-spatial attributes changed during the history of reindeer herding in northernmost Finland? And how can NCT be applied in illustrating the modification of the environment (niche) by multiple niche constructors?

### Niche Construction Theory (NCT)

The conceptual approach of NCT is based on evolutionary biology, which emphasizes the capacity of organisms to modify natural selection in their environment and thereby act as co-directors of their own and other species’ evolution (Odling-Smee et al. 2003:419; Laland et al. 2008:549; Odling-Smee et al. 2013:3). Where classical evolutionary theory sees the organism as the key that has to fit into the environment’s lock, or views adaptations as solutions to the problems posed by the environment, NCT asserts that organisms and their ecological niches co-construct and co-define each other (Lewontin 1983). Put in classical terms, organisms adapt their environment rather than adapt to it (Laland and Sterelny 2006).

NCT stresses that organisms frequently modify the environments of other organisms that share the environments with them. An organism has as an ability to alter its environment by processes such as migration, dispersal and habitat selection. In short, an organism chooses an environment, modifies it and creates it, or destroys a habitat and resources for other living creatures; these modifications may then become evolutionarily significant (Sterelny 2001:333; Odling-Smee et al. 2003). This approach addresses the significance of development in which the evolutionary process depends not only on the processes of natural selection and genetic inheritance, but also on the process of ecological inheritance through niche construction (Lewontin 1983;Sterelny 2001:333; Laland and Sterelny 2006; Laland et al. 2008:550).

We use the concept of “construction” when applying NCT as a conceptual framework in describing the changes of the past and present. We re-interpret biological discussions of cause-and-effect relationships in order to understand the multiple variables involved and their interrelationships. The results from studies of biology and ecology can be regarded as giving “scientific messages from a northern biotic world” their own legitimacy as scientific language. These messages allow us to combine scientific data with the IK of Sámi herders as part of constructing reindeer behaviour and human actions. The IK of Sámi herders in this study refers to knowledge that encompasses narratives as well as analytic discussions. Part of this knowledge is connected to everyday practice which includes nonverbal actions and context-situated learning (Berkes 1999; Ingold 2000; Lave and Wenger 2001; Polanyi 2002; Helander-Renvall 2008; Saijets and Helander-Renvall 2009; Riseth et al. 2010; Näkkäläjärvi 2013).

### Material and methods

**Figure 1:**
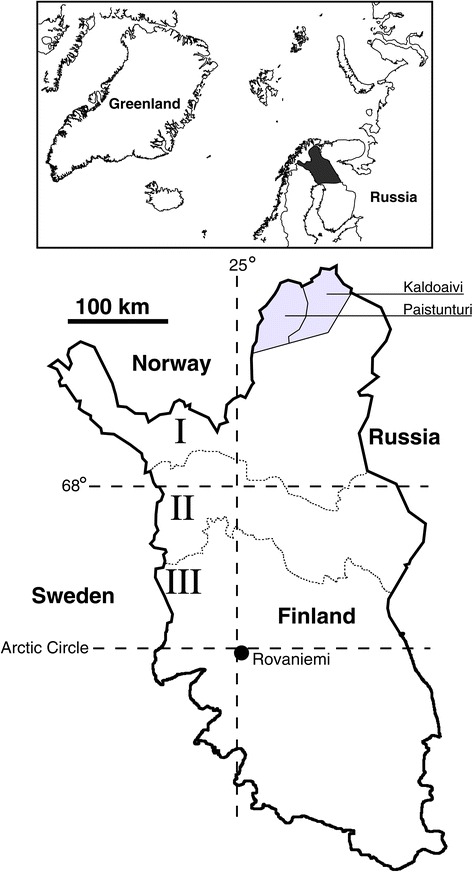
**Location of the herding co-operatives studied and the borders of the area known as the Sámi reindeer herding area (I), Special reindeer herding area (II) and Other reindeer herding area (III) in Finland.**

The study has been conducted in two northern (Fell) Sámi (Sapmi) herding co-operatives in Finland, Kaldoaivi and Paistunturi (Figure 1). In the Sámi herding area, special attention should be paid to safeguarding reindeer husbandry against encroachment by other land uses. At present there are no other major land uses competing with reindeer herding in the Kaldoaivi and Paistunturi co-operatives, but there is a threat that mining activities will start in the future, as licenses have been granted to companies for test drilling. Both co-operatives belong to the municipality of Utsjoki, which has 1298 inhabitants and is the only municipality in Finland where the Sámi are a majority. The Sámi language spoken there is northern Sámi (Population Register 2012). Most of the people belong to families whose livelihood is based on reindeer herding, including meat processing; salmon fishing; and/or tourism.

**Figure 2:**
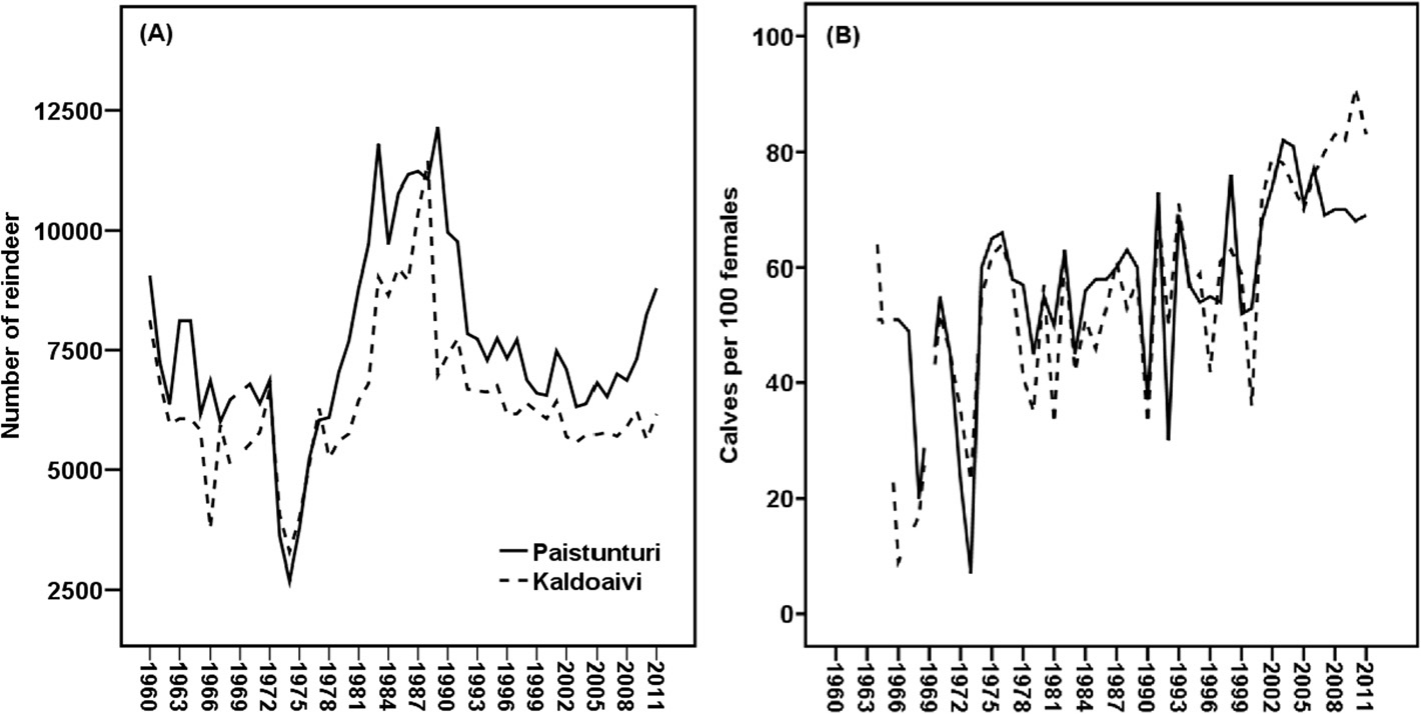
**Number of reindeer (A) and calving percentage (calves per 100 females) (B) in Paistunturi and Kaldoaivi during the period 1960–2012 (RHA** 2014 **).**

The vegetation of the study area is characterized by subarctic mountain birch woodlands, treeless heaths and barren fell tops (Oksanen and Virtanen 1995). The area of lichen pasture per reindeer is 11.07 ha in Kaldoaivi and 12.50 ha in Paistunturi, and the area of shrub, deciduous, and herb pasture per reindeer is 19.89 ha and 22.37 ha, respectively. The impact of infrastructure on the total surface area is 3.10% (Paistunturi) and 4.57% (Kaldoaivi) (Kumpula et al. 2009). The reindeer numbers and calf percentages of the co-operatives are presented in Figure 2. The largest permissible number of reindeer is 5300 in Kaldoaivi and 6300 in Paistunturi (RHA 2014). The reindeer density in Kaldoaivi is 2.38 and in Paistunturi 2.17 reindeer per km^2^. Reindeer can move freely and there are no round-ups to earmark calves during summer. After the autumn/winter round-ups, at which meat is sold, reindeer are either separated and placed in *siida/family* pasture areas for the rest of the winter (Kaldoaivi) or herded using hay by each siida during the late spring (Paistunturi).

The data used consist of both semi-structured interviews and discussions. A total of 15 Sámi reindeer herders were interviewed between 2007 and 2011. We used a snowball sampling technique for gathering informants in which we identified the managers of the co-operatives, who then provided the names of other informants, opening up an expanding web of contacts. Thirteen of the herders were males (M1-13), and two females (F1-2). The age of the herders ranged from 30 to 75 years. All informants were full-time herders and were members of the traditional Sámi siida system (Vuojala-Magga 2012; Helander-Renvall 2014). Three of the herders were retired, but their family members were active in herding. Four interviewees were either former or present managers of the co-operative. Most of the informants earned supplementary income from meat processing, tourism (guided activities or accommodation), fishing and/or hunting. Guided activities include salmon tourism, that is, taking tourists by boat to fish in the summer.

The first nine interviews were conducted by an anthropologist (Terhi Vuojala-Magga) and a biologist (Minna Turunen) in the period 2007–2008 in order to collect background data and formulate more specific aims for the study. We used maps of pasture surveys (Kumpula et al. 1997, 2009) to locate siidas, reindeer migration routes, regions of mountain birch forest destruction and other places of importance to herding. The herders were asked about the characteristics of their pastures and herding practices as well as the significance for herding in the present and the past of mountain birch forests and their destruction by moths. Another set of questions was presented relating to the effect of extreme weather and climate change on pastures and herding practices (Vuojala-Magga et al. 2011). The herders were asked questions such as: What is your pasture land like? What kind of herding system do you have? What has been the impact of autumnal and winter moth outbreaks on pastures and herding? What were the impacts of the autumnal moth outbreaks in the 1960s on pastures and herding? What is the importance of weather for pastures and herding work?

The second phase of fieldwork was conducted in 2011 by an anthropologist and included eight interviews centred on the contemporary scientific publications on moths, reindeer and mountain birches (e.g. Klemola et al. 2003, Klemola 2009, Tømmervik et al. 2009, Ammunét 2011). The results put forward in these publications were presented in popular form to the herders, who were asked key questions such as: What do you think if outbreaks of both autumnal and winter moth become more common as the climate gets warmer? What do you think about the impact of global warming and reindeer on the growth of birch trees? What do you think of the future of reindeer herding? What do you think of the future of the mountain birch forests in your area?

The interviews were conducted in Finnish, tape-recorded, transcribed and analysed by both authors. This study also draws on the position of one of the authors (T. V-M) as a member of a Sámi reindeer-herding family in the village of Kuttura in Hammastunturi. The interactions between the researchers and informants were thus not only based on interviews but on a mutual trust due to shared knowledge of reindeer herding and Sámi kinship systems. In this respect, the research follows the principles of radical empiricism, which emphasizes that knowledge and understanding come primarily from lived experiences based on everyday life and work, in the present case these being work within the herding community (James 1976; Jackson 1989; Rosaldo 1989).

In addition to the interviews and long-term participation in daily herding work and field observations (T. V-M), we used historical documents; professional and scientific literature on Sámi reindeer herding, land use and human population movements; statistical data on reindeer populations (RHA 2014) and contemporary biological studies.

## Results

### The nomadic era: niche construction from the end of the 19th century to the beginning of the 20th century

The interaction of human beings with wild reindeer and the animals’ grazing and trampling behaviour has shaped the northern ecosystems since the last ice age (Suominen and Olofsson 2000:235). Nomadic Sámi herding emerged in the 17th century (Näkkäläjärvi 2007:40), and was based on pasture rotation from the coastal summer lands of Norway to inland areas of Finland-Sweden or, after Finland was ceded to Russia by Sweden in 1809, Finnish Russia (the Grand Duchy of Finland under the Russian Empire). In difficult winters, marked by hard snow, thick snow or icy ground, reindeer exhibited an emerging characteristic of seeking forage outside of the normal pasture rotation. People used to say: “*After a good lemming year* there *will be a bad year for reindeer* ”(M1-M13, F1-2). The reindeer “*broke loose*” and went into forests with a rich availability of arboreal lichens or onto fell slopes with “wind-whipped snow” (Gibson 1979; Vuojala-Magga et al. 2011; Turunen and Vuojala-Magga 2014). Animal behaviour like this can be seen as a temporal act of habitat selection resulting in an enlargement of the animals’ niche. However, harsh conditions increased winter mortality and/or reduced the reproductive rate of the weakest reindeer; in early winter these were the young rutted bulls, thereafter the one-year-old calves and, in late winter, the pregnant females.

In 1852 the border between Norway and Finland was closed and the human-reindeer migration routes from the coast to the forest areas were shut down (Itkonen 1948). Closing the border was a lengthy process. The official reasons given for doing so were that high numbers of reindeer migrated from Norway to Finland, damaging crops and lichen pastures, and that forest reindeer owned by Inari Sámi herders were joining the herds going back to Norway (Itkonen 1948; Enbuske 2008; Jouste 2011). As a result, many families moved from Norway to Sweden with their herds to continue the winter migration to Finland, as the border between Sweden and Finland was still open. The niche based on pasture rotation was re-arranged among the Sámi families, who had used lands in Norway and Finnish Russia. However, grazing pressure increased in some areas in northern Sweden, which led to a deterioration of relations between Sweden and Norway. Eventually, in 1883, a law regulating human-reindeer movement between the countries was passed, and by 1889 the border between Sweden and Finland was also closed (Itkonen 1948; Näkkäläjärvi 2007: 42–54; Jouste 2011; Koch and Miggelbrink 2011).

In spite of the closure of the border, the nomadic Sámi of Utsjoki herded their reindeer to Norway until the 1860s (Itkonen 1948). An old herder recalls the old pasture rotations: *During those years when there were no fences separating co-operatives, reindeer foraged in their natural ways – there were rutting places, calving places and winter areas. And once the sun began to rise, the reindeer rose up on the mountains; people moved with the reindeer, followed them and remained with the herd. In summer time reindeer grazed in the coastal area of Varangerbotten (in Norway) and later (winter) went all the way to the Saariselkä forest area (in Finnish Russia). The main thing was not to mix up different herds*” (M1). Over time pasture rotation diminished and herders ended up with smaller pasture regions in Utsjoki, in Finnish Russia (Itkonen 1948). This meant that reindeer were herded nearly 12 months per year on the same pasture lands. The carrying capacity of the land was exceeded (Jouste 2011:47). Between 1878 and 1882 there was a dramatic decrease in the reindeer population, from 31,683 to 19,633 animals (Jouste 2011:43). In 1898, reindeer herding in Finnish Russia was organized in a system of geographically defined co-operatives and the old nomadic routes were discontinued by the end of the century (Kortesalmi 2007).

The border closures caused drastic changes within the herding communities. The decrease in pasture land reduced the availability of winter forage. The Russian Czar’s act mandating sustainable use of reindeer pastures brought out the characteristics of reindeer in a new light; grazing and trampling negatively affected niche construction and reindeer became key constructors of the niche for the time being. Neither animals nor herders had possibilities for adaptation, but rather were forced to seek new land in the more southern, forested areas of Finnish Russia; they sought to enlarge their niche, because there was still room for new people and herds farther south (Jouste 2011).

### Moth outbreaks in the old days

Although there have been massive moth outbreaks throughout the past centuries, hardly any old documents on the outbreaks exist from Finnish Russia. There were shifts in the elevation of the birch tree line already during the 19th and early 20th century in Abisko, Sweden. There are clear indications that the combination of mass moth outbreaks and reindeer browsing has destroyed mountain birch forests since the beginning of the Holocene (Holtmeier and Broll 2006). In the years 1902–1903, moth outbreaks caused severe damage to the fell vegetation. Documents from the 1920s indicate that Sámi herders gradually abandoned campsites in the affected areas (Emanuelson 1987; Cairns and Moen 2004; Van Bogaert et al. 2011:918–917).

In northern Utsjoki, mass outbreaks of autumnal moths have been documented for 1844, 1905, 1909, 1927, 1957, 1965 and 1966 (Holtmeier et al. 2003). One herder recollected the following from the 1950s: “*I remember this time, I was less than 20 years old… My late father told me that the area was eaten some 35 years ago, and there were only stumps of this height left; nothing had grown afterwards. This was around 1920–1930, and no new trees have grown back in this area*” (M2). This story most likely refers to the moth outbreak of 1927.

The interviews allow us to evaluate construction of the niche, with its temporal and spatial dimensions. The main driver of the spring migration of reindeer is the need to have a peaceful, dry and mosquito-free calving site with good access to fresh green forage. If a destroyed birch forest was located at a calving site or was on the reindeer’s route to the summer pasture, the herds still headed to these sites, led by mature females (Turi 2011). The insect harassment made the reindeer head uphill on the fells to where there were fewer mosquitoes, and the herders then had an easier time managing the animals, which were said to be “*softened by mosquitos*” . The significance of permanent summer pasture areas comes out clearly in the herders’ accounts: “*There were no fences on the fell, just smokes, and reindeer came to sleep by the smokes during the day, when the mosquitoes were worst. In daytime people just walked around among reindeer and calves and cut earmarks. At night time the reindeer left; it was not hot, there were not that many mosquitoes and they went to eat all together*” (M3).

A few years after a mass moth outbreak, wavy hair-grass (*Deschampsia flexuosa*) colonized the soil around the birches due to the combined impact of nutrients from moth larva faeces, increased light reaching the ground after the death of the birch canopy and decreased competition given that the larvae also destroy the dominant dwarf shrubs (Rybråten and Hovelsrud 2010). As a herder from Kaldoaivi explained: “*It went like this; first there was lichen, then autumnal moths ate the buds, then there was grass, this height; the grass killed the lichen. Our reindeer were really fat; then the grass ran out and mosses came, which meant no more nutrition* (M6). As new forage appeared, the reindeer had no need to make any immediate changes. After 4 to 10 years, however, the disappearance of the grass affected the movements of the female reindeer and each year they had to enlarge their foraging areas. Changing the pasture rotation is a slow process, as seen in Abisko, Sweden, where the damaged pastures were abandoned slowly (Van Bogaert et al. 2011). Both the moth outbreaks and border closures were more serious and permanent incidents than the occasional harsh winter weather. Negotiations were conducted and new arrangements made between the siidas, which were forced to change their nomadic routes. In the worst case, the herders started to lose their reindeer for good. Reindeer took on the role from the moths as key constructor of the shared niche and modified their own forage resources by depleting them. Moths first destroyed the primary grazing lands of reindeer, after which the reindeer, as flexible herbivores, carried on the destruction by browsing the damaged birch trees and later grazing on the new grass that appeared. Because parts of the niche were abandoned, the reindeer were forced to extend their pasture rotation to new lands. Here one can see a niche being constructed in a positive way by the action of an animal itself.

### Perceptions of moth attacks during disastrous years of the 1960s

Until the 1950s reindeer were herded intensively in northern Finland (Kortesalmi 2007). During disastrous years reindeer tended to expand their niche onto new pastures, as they had before. Tensions emerged between the members of different co-operatives. From the perspective of forest herders in the southern parts of the herding area, the big northern herds were a threat in forest areas, as the herders were afraid of losing their reindeer on the fells during late spring. Between 1960 and 1990 the present system of fences between co-operatives was introduced. The niches became “permanently” constructed, which mostly benefitted the small reindeer owners in forest areas. During hard years fences prevented reindeer looking for new forage or acting in enlarged niches. At the same time, extensive herding had replaced intensive herding because of “the 1960s snowmobile revolution” (Pelto et al. 1968; Helander-Renvall 2008; Näkkäläjärvi 2013). This meant that reindeer moved inside the fenced co-operatives without people herding them (Helle and Jaakkola 2008; Vuojala-Magga et al. 2011; Turunen and Vuojala-Magga 2014). During this era of fenced and permanent herding co-operatives, diminished land use, severe winters and moth attacks, the negative ecological inheritance that emerged as a selection pressure favoured the fittest reindeers.

Moth outbreaks in the 1960s destroyed about two-thirds of the mountain birch forests in northernmost Finland (Tenow 1972). For example, in the year 1964–65 in Utsjoki, autumnal moths defoliated 1350 km^2^. One of the herders described the situation as follows: “*They just ate everything, the ground vegetation, too. It’s just open, from here all the way to the north, to Pestausselkä 10 km…[] There is nothing but open, flat (landscape) once you drive some 20 km to Inari. There used to be birches before. These green caterpillars, 2 or 3 on each leaf - just count how many there were in each tree. It was just … you should just listen to it*.” (M6). According to herders, “*the oddest thing was the green leaves in the autumns; after the moth outbreak if the weather is warm enough, green leaves will sprout*.” (M7, F1). These late-summer leaves attracted reindeer to browse again. Wavy hair-grass, which reindeer grazed on (Rybråten and Hovelsrud 2010:323), grew well for many years before it disappeared and the landscape changed into open land with black soil, like “*the surface of the moon*”. (M1). As one of the herders noted: “*The foliage destroyed by autumnal moth outbreaks in the 1960s has not yet recovered; what we see is a biological death; it has been a poison* (M8). What had been forests became open tundra (Lehtonen and Yli-Rekola 1979; Haukioja et al. 1988; Klemola et al. 2003:354). Still today rotten birch stumps or peaty hummocks signal former birch habitats (Holtmeier et al. 2003). The birch defoliation in 1960 was most probably connected to the combined response to mass moth outbreaks, reindeer browsing, rotting roots and cold summers (Lehtonen and Heikkinen 1995; Holtmeier et al. 2003; Holtmeier and Broll 2006; Holtmeier 2012:7; Huttunen et al. 2012, 2013).

There has been discussion regarding the role of reindeer in determining the location of the birch tree line. Whether reindeer grazing should be considered a disturbance for fell areas is a complex issue (Kallio and Lehtonen 1975; Helle and Kojola 1993; Lehtonen and Heikkinen 1995; Oksanen et al. 1995; Helle et al. 1998; Helle 2001, den Herder and Niemelä 2003; Holtmeier et al. 2003; Holtmeier and Broll 2006, 2007, 2009; Riipi et al. 2005; Holtmeier 2007, 2012). What is more, there is not much scientific evidence for the recovery of forests after the moth outbreaks in the 1960s, because high reindeer numbers caused considerable grazing pressure on birch forests. In Kaldoaivi and Paistunturi, reindeer numbers rose rapidly from their minimum in 1974, reaching their maximum in the period 1987–1988 (RHA 2014) (Figure 2).

Since the beginning of the 1990s, herding in Kaldoaivi and Paistunturi has faced new changes. Summer round-ups, in which calves were earmarked, have been discontinued and this has protected lands and increased the well-being of calves. The composition of herds has changed as well. The number of adult males has decreased, because meat sales are based on the slaughter of male calves; this protects the winter pasture lands (Heikkinen 2006). Finally, in the 1990s herders changed the free-ranging system to one of spring herding with hay. Owing to this, the herds are more resistant to harsh winters and the rest of the delicate lichen cover can be protected (Vuojala-Magga et al. 2011). Since the 1960s, humans have become indirect key constructors by establishing permanent niches – fenced-in co-operatives - on a smaller scale than ever before. People had no experience-based knowledge of seasonal pasture rotation inside the fences. This system relied on the reindeer to find forage in any conditions. Access to a temporally extended niche was not possible. Moths and reindeer became key constructors one after another during the years of mass moth outbreaks. As there was no old solution for overcoming the hard times, herders had to apply the traditional systems of herding and knowledge of the herd’s behaviour/social system in new ways with new technology. This can be seen as an adaptive process in response to new ecological inheritance in a specific, restricted environment. The herders gradually took on the role of niche constructors in tandem with their animals. The co-operation between herders and reindeer can be seen as increased domestication of reindeer (Helle and Jaakkola 2008; Vuojala-Magga et al. 2011; Turunen and Vuojala-Magga 2014).

### Global warming as a factor in niche construction

According to the IPCC (2013), the increase in the global mean surface temperature for the period 2081–2100 relative to 1986–2005 is projected to be in the range of 0.3 to 4.8°C. The Arctic region will warm more rapidly than the global mean. Due to warming, both coniferous and mountain birch forests are expanding northwards and upwards on the fell slopes. Fast-growing graminoids, herbs and shrubs may replace slowly growing lichens (Kullman and Öberg 2009; Turunen et al. 2009; CAFF 2013). Similar observations have been made by herders: “*There has been enormous growth of birches. The trees and leaves are so dense on the riversides that you can no longer see the rivers* (M1, M8). *Those areas (destroyed) in 1964 have been regenerated and are now dense with birch trees* (M1).

**Figure 3:**
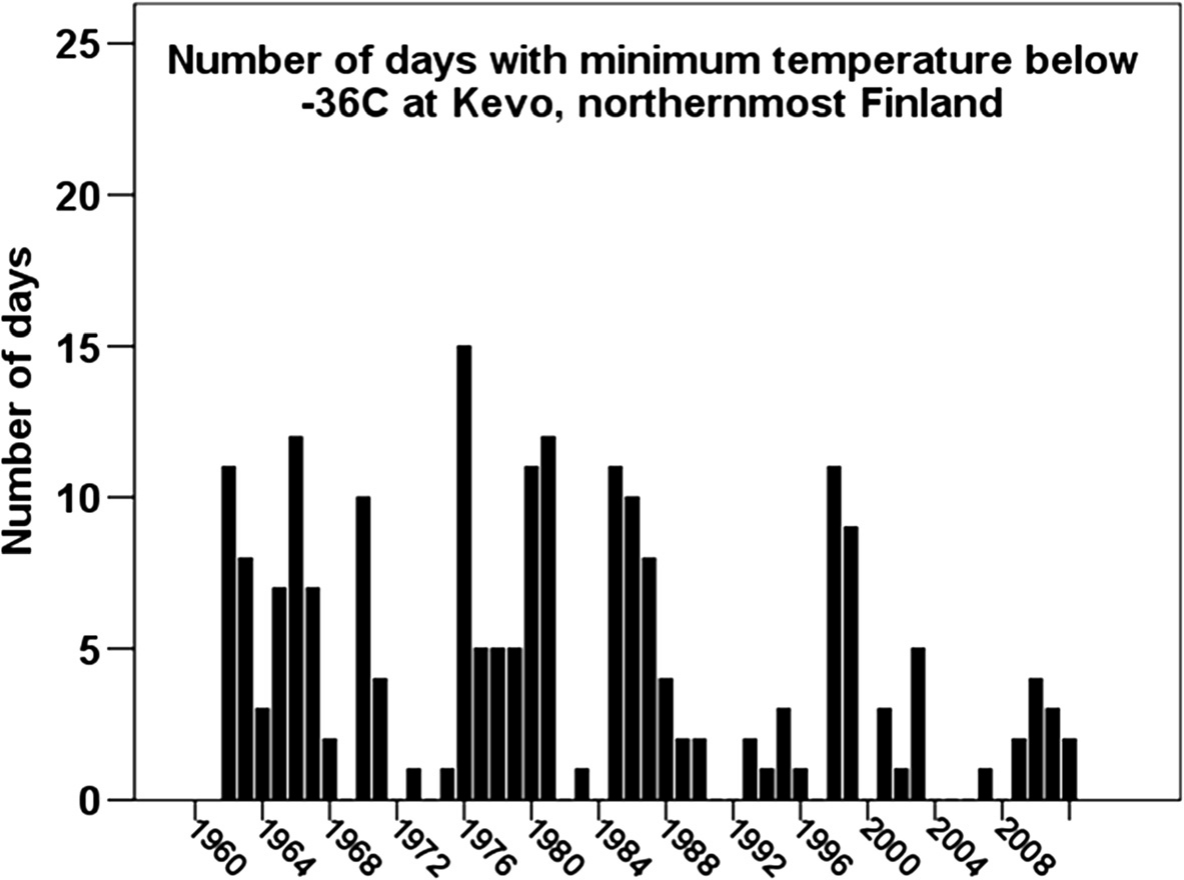
**Number of days with minimum temperature below −36°C at Kevo, Finland (FMI** 2013 **).**

It is predicted that the combined occurrence of mass outbreaks of autumnal moths and a new invasive species of winter moth will be more frequent, because milder winters will increase the survival rate of moth eggs (Neuvonen et al. 1999; Ayres and Lombardero 2000; Logan et al. 2003; den Herder et al. 2008; Klemola 2009; Ammunét 2011, Ammunét et al. 2012). For example, at Kevo, in northernmost Finland, the number of days with a minimum temperature below −36°C (critical for egg survival) decreased during the period 1962–2013 (FMI 2013) (Figure 3). The winter moth has already now expanded its range to continental areas of northern Finland (Jepsen et al. 2008; Klemola 2009; Karlsen et al. 2013). In the years 2006–2008 (2009) the destruction of birch forest in the Nuorgam and Polmak areas of Utsjoki was caused by winter moths. It was a new experience for herders: *“I went to catch whitefish with my cousin. I was driving in front of him, and I stopped, and asked, ‘Can you see … that the ground and soil are alive?’ There were these balls the size of a human fist – worms stuck together – awful balls full of life; they were greenish worms and just full of life. They had just eaten all the birches, and it was (as late as) September* (M2).

Studies on the effects of warming and reindeer browsing on mountain birch growth have produced conflicting results (Kallio and Lehtonen 1975; Lehtonen and Heikkinen 1995; Oksanen et al. 1995; Helle et al. 1998; Suominen and Olofsson 2000; Helle 2001, den Herder and Niemelä 2003;Cairns and Moen 2004). Helle (2001) claims that where regeneration of mountain birch is inhibited by herbivory, the forest will gradually change into tundra. Where there is intensive browsing by reindeer, birches exhibit a typical apple tree–like shape, with no lower branches (e.g. Kallio and Lehtonen 1975; Oksanen et al. 1995; Helle et al. 1998; Suominen and Olofsson 2000:240; Holtmeier and Broll 2006, 2009). A hypothesis regarding the removal of the “lichen barrier effect” maintains that heavy reindeer grazing could lead to the decline of the lichen cover, which would then make upward migration of the tree line possible (Helle and Aspi 1983; Tømmervik et al. 2004, 2009). This occurs because lichens can inhibit seed germination and growth of seedlings and sprouts through chemical and physical impacts (Brown and Mikola 1974). On the other hand, reindeer herbivory could act as a buffer countering the rise of the tree line due to warming (Helle and Aspi 1983; Virtanen et al. 2003; Tømmervik et al. 2004; Bond 2006; den Herder et al. 2008; Kaarlejärvi 2014). A recent study indicates that large mammalian grazers may slow down vegetation changes and that moderate grazing pressure could protect small tundra forbs from being outcompeted by taller plants under climatic warming (Kaarlejärvi 2014). The connection between reindeer grazing and moth outbreaks is a delicate issue for herders. The ways in which this topic is addressed depend on the nature of the livelihood. Herders who earn a substantial proportion of their income from other sources, for example tourism, might attribute the signs of erosion in fell areas to high numbers of reindeer: “W*e only need to have a couple of severe years…before desertification starts”* (M9).

When discussing future birch destruction, herders recalled their past experiences. They know that reindeer are able to use 200 to 300 plant species for nutrition (Warenberg et al. 1997) and that, in addition to their mountain birch forests, the regions of Kaldoaivi and Paistunturi herding co-operatives have vast wetlands: “*There will be always green somewhere, like willows, and birch trees among willow areas are always undamaged; there is grass in those areas. Once damage occurs, the reindeer seek new green areas where they can eat a lot of different types of green plants – like blueberry and whatever”* (M1). “*If there is pressure on the soil, the number of reindeer will decline and then small reindeer owners will be out first*” (F2). This means that with higher reindeer numbers there is a greater possibility to sustain the livelihood. “*We might well face a hard situation and, indeed, we are getting ready for this – nature has taught us…. We have been prepared for this type of situation; we will use various means to overcome and cope and get through the hardships. Those who do not bother to work will drop out (of reindeer herding)* (M1).

## Discussion and conclusions

**Figure 4:**
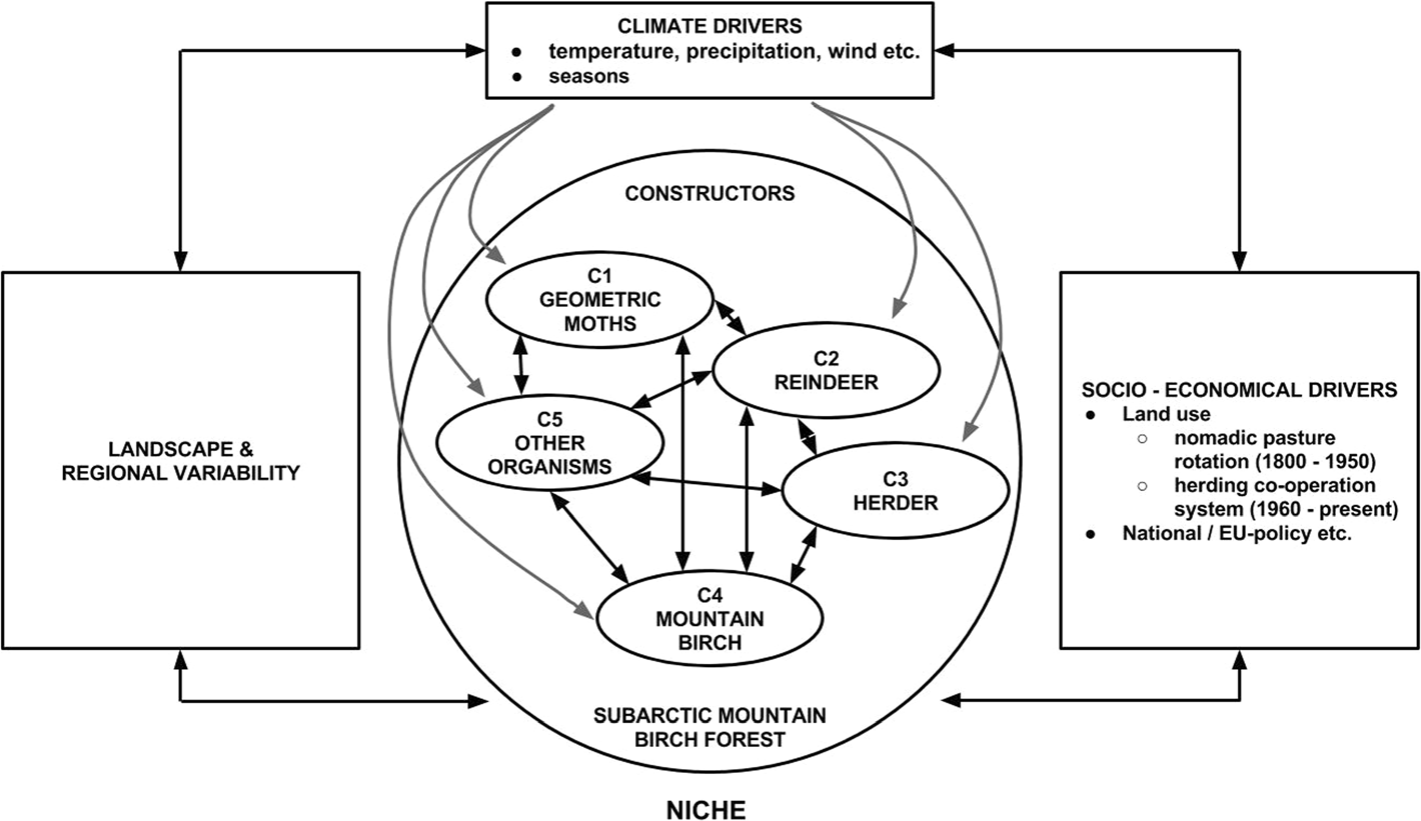
**A simplified model describing the main relationships and interactions between the niche constructors (C1-C5) within the subarctic mountain birch forest, as well as the impacts on the niche of climatic and socio-economic drivers and landscape and regional variability.** The role and importance of niche constructors has varied, for example, due to the reindeer herding system in use and national and European Union policies. Geometer moths include both the autumnal moth and winter moth. Other organisms include predators, diseases, parasitoids and reindeer forage plants (e.g. lichens, willow, wavy hair-grass).

Geometer moths, reindeer, herders, mountain birch and other organisms have constructed a niche in various ways in Finnish Russia and, later, independent Finland over the past 150 years (Figure 4). In this study, the niche constructors are first looked upon as “equal constructors” of a shared niche. As the process of change unfolds in their niche, we see their roles changing from constructor to key constructor. The role and importance of these niche constructors were different during nomadic pasture rotation (19^th^ century – 1950) than under the herding co-operative system (1960-present). From our perspective NCT is not only positive, but opens up the human role as a final key constructor, which can be either negative or positive in the long run.

In the 19^th^ and early 20^th^ centuries, the nomadic annual cycle of reindeer and herders dominated construction of the niche and its temporal and spatial dynamics, which can be regarded as positive niche construction. The accessibility of forage determined the scale of reindeer movements, and thus the affordances of the spatial dimensions of the niche. The heterogenic composition of the herd (e.g. gender, age, size) for many generations (Tyler et al. 2007) increased the dimensions of the niche. The role of mature and experienced reindeer, with their emerging characteristics, has been crucial during difficult times. The animals’ sense of smell, ability to communicate and strength are central characteristics, for example, when digging for lichens through hard snow or looking for epiphytic lichens or fresh green forage plants (Vuojala-Magga et al. 2011).

Moth outbreaks have resulted in far-reaching and almost permanent changes in niche construction by changing the conditions of other populations within the mountain birch forest communities. By modifying their own life-worlds, both moths and reindeer have modified the selection pressures on their descendants via ecological inheritance. Reindeer, together with cyclic moth outbreaks, have become key constructors of the niche. In addition, the past forty years have seen a change in the role of the human being (reindeer herder) from indirect to active niche constructor. In the herding co-operative system that has been used since the 1960s, each co-operative can be viewed as a micro-niche with its own, independent herding system and as benefitting from the local microclimate, topography, vegetation and the siida system (Helle and Jaakkola 2008; Vuojala-Magga et al. 2011; Turunen and Vuojala-Magga 2014). In Kaldoaivi and Paistunturi, new adaptive ways of herding were adopted, such as autumn calf-markings, slaughter of male calves, parasite medication, as well as new pasture rotations that included intensive herding with hay during the spring months. These measures included a new type of flexibility, controlled spatiality and specialization, which were adopted for the sake of animal well-being and sustainability in light of the diminished land use. Reindeer eventually became tame, as they had been during the nomadic era (Vuojala-Magga et al. 2011).

Global warming - as a factor in niche construction along with the emerging characteristics of moths and reindeer - can be viewed in various ways. Firstly, the combination of two moth species and reindeer as key constructors might destroy birches in larger areas than earlier. However, complete destruction is difficult for herders to imagine, because there is no sign of such a process occurring. Were this to occur, the herders assume that it would lead to a radical reduction in the number of reindeer and herders. This can be seen as one of the results of negative ecological inheritance in NCT. Secondly, due to warmer and longer growing seasons birch growth will increase and defoliated areas may recover faster than before (Huttunen et al. 2012). Herders have observed that the defoliated areas of 1960s have partly started to recover. If this process continues, the niche will become more heterogenic than before. Thirdly, reindeer may act as a catalyst for balancing the location of the treeline by keeping up a buffer against invasive species (Kaarlejärvi 2014). The same idea can be seen another way around: reindeer could help seed more species by moving around the fell areas. These perspectives highlight the role of reindeer as a continuous key constructor of the niche in the context of warming. From the perspective of herders, warming is seen as a long-term change in nature. The prehistoric pine tree roots on fells tell herders about the warmer climate during the Holocene: “*Here the present distress is just life, and something will always grow. It just cannot die out; something green will always grow. We have managed here since 9000 BC, and I suppose there was some green then, too. I think we will survive*.” (M1) Warming as such is not a problem if it means increased precipitation and growth; however, drought and hot summers are hard for reindeer, and vegetation growth on the high, dry mosquito-free terrain will still be endangered by reindeer trampling.

Permanent changes in the niche caused by the closure of borders over the second half of the 19th century ended the reindeer migration between Finland and Norway and Finland and Sweden and caused extreme grazing pressure on lands, with reindeer becoming a key constructor of the niche (Caballero et al. 2007:406; Jouste 2011). The main reason for building fences between the co-operatives after the 1960s was a change in herding technique; the former intensive herding was replaced by extensive herding due to the use of snowmobiles (Pelto et al. 1968; Helander-Renvall 2008; Näkkäläjärvi 2013). The reindeer ranged loose most of the year as the fences prevented herds from becoming mixed, and the animals became more feral (Helle and Jaakkola 2008; Vuojala-Magga et al. 2011; Turunen and Vuojala-Magga 2014). Going back to the old type of nomadic migration routes by opening up the border between Finland and Norway has been seen as a good solution for Norwegian reindeer in Finnmark when difficult snow conditions pose a threat (Riseth et al. 2004; Caballero et al. 2007; Callaghan et al. 2011). However, on the Finnish side, the reindeer in the Paistunturi and Kaldoaivi co-operatives enlarged their spatial range *temporarily* during hard times*:* they *“broke loose*” from the tight control of the herders. The invasion of hay fields in Finland by Norwegian reindeer was *constant,* occurring year after year: “*Nowadays in Norway there are rules stipulating when one can drive one’s reindeer to the summer and winter herding regions. Today the herds there are so large that nothing is enough for them*”(M1, Koch and Miggelbrink 2011). This and the fact that new border fences are being built between Finland and Norway are some of the reasons why Finnish Sámi herders have expressed critical views of opening up the border when trans-national reindeer management has been discussed (M1-M13, F1-2).

NCT is not only about spatiality but about the actions and emerging characteristics of organisms as niche constructors that produce ecological inheritance and fitness. Micro-niche construction has taken place for nearly forty years among reindeer, moths and humans in northern Finland. Reindeer herding is modifying the shared niche as, for example, forage for the late-spring herding is now produced from hay fields of former cattle ranches (Turunen and Vuojala-Magga 2014). In this way, herders as niche constructors are keeping up the traditional biodiversity of riversides. Furthermore, reindeer have become familiar with the spring herding practice and there is no need for reindeer in Finland to “break loose” as long as their nutritional needs and fitness are secured. From the perspective of NCT, a transnational herding system should offer better fitness to the reindeer of the Finnish Sámi; however, at the moment this would not be case.

Niche construction theory (NCT) and the importance of niche construction have been subjected to critical evaluation. For example, there has been some debate over whether niche construction is an evolutionary process, how natural selection leads to organismal adaptation and whether niche construction and natural selection are of equivalent explanatory value (Scott-Phillips et al. 2013; Matthews et al. 2014). In this study, we used NCT as a theory to describe the modification of a subarctic environment by niche constructors - geometer moths, reindeer, herders and mountain birch (Figure 4) - and not merely to challenge other theories, for example, standard evolutionary theory. Instead of having the ultimate focus on selection pressure, the focus of NCT is on local environmental change and active constructors in producing ecological inheritance, which may influence the selection pressures underlying evolutionary processes.

NCT, applied here as a model that accommodates multiple variables, offers a feasible approach for looking at the interrelationships of different organisms or actors, for example, flora, fauna or human beings, not only from the human perspective but also from a shared perspective of different organisms (Figure 4). In this respect NCT comes closer to the shared ontology of the human-animal relationship (Ingold 2000). The strength of NCT lies in its context-situated approach; for example, the shift from one key constructor to another evolves in the context of multiple variables. The weakness of NCT when applied in re-constructing a niche and its temporal and spatial dynamics is that the multivariable construction does not produce in-depth information about individual actors. For example, the problem of the systems of fences between the countries and between the herding co-operatives in Finland is largely a facet of the human perspective, that is, the governance or politics of reindeer herding, and therefore does not fall with the scope of the present study.
